# Portfolio Optimization: A Neurodynamic Approach Based on Spiking Neural Networks

**DOI:** 10.3390/biomimetics10120808

**Published:** 2025-12-02

**Authors:** Ameer Hamza Khan, Aquil Mirza Mohammed, Shuai Li

**Affiliations:** 1 School of Artificial Intelligence (AI), Taizhou University, Taizhou 318000, China; ahkhan@tzc.edu.cn; 2 Smart City Research Institute, The Hong Kong Polytechnic University, Hong Kong; 3 Department of Computing (COMP), The Hong Kong Polytechnic University, Hong Kong; aquilmirza.mohammed@polyu.edu.hk; 4 Faculty of Information Technology and Electrical Engineering (ITEE), University of Oulu, 90100 Oulu, Finland

**Keywords:** portfolio optimization, spiking neural networks, constrained optimization, real-time computing, cardinality constraint, computational finance

## Abstract

Portfolio optimization is fundamental to modern finance, enabling investors to construct allocations that balance risk and return while satisfying practical constraints. When transaction costs and cardinality limits are incorporated, the problem becomes a computationally demanding mixed-integer quadratic program. This work demonstrates how principles from biomimetics—specifically, the computational strategies employed by biological neural systems—can inspire efficient algorithms for complex optimization problems. We demonstrate that this problem can be reformulated as a constrained quadratic program and solved using dynamics inspired by spiking neural networks. Building on recent theoretical work showing that leaky integrate-and-fire dynamics naturally implement projected gradient descent for convex optimization, we develop a solver that alternates between continuous gradient flow and discrete constraint projections. By mimicking the event-driven, energy-efficient computation observed in biological neurons, our approach offers a biomimetic pathway to solving computationally intensive financial optimization problems. We implement the approach in Python and evaluate it on portfolios of 5 to 50 assets using five years of market data, comparing solution quality against mixed-integer solvers (ECOS_BB), convex relaxations (OSQP), and particle swarm optimization. Experimental results demonstrate that the SNN solver achieves the highest expected return (0.261% daily) among all evaluated methods on the 50-asset portfolio, outperforming exact MIQP (0.225%) and PSO (0.092%), with runtimes ranging from 0.5 s for small portfolios to 8.4 s for high-quality schedules on large portfolios. While current Python runtimes are comparable to existing approaches, the key contribution is establishing a path to neuromorphic hardware deployment: specialized SNN processors could execute these dynamics orders of magnitude faster than conventional architectures, enabling real-time portfolio rebalancing at institutional scale.

## 1. Introduction

Portfolio optimization with transaction costs and cardinality constraints presents a fundamental challenge in quantitative finance: determining an optimal asset allocation that balances expected returns against risk while respecting practical trading constraints. The classical Markowitz mean-variance framework [1] provides an elegant analytical solution for unconstrained continuous portfolios, but real-world requirements—transaction fees [2], regulatory limits on diversification, and the need for sparse holding—transform the problem into a mixed-integer quadratic program [3] that resists efficient solution. Exact branch-and-bound methods [4] deliver provably optimal solutions but scale poorly with problem size, while metaheuristic approaches [5,6,7] sacrifice optimality guarantees for computational speed without offering a clear path to further acceleration.

Recent theoretical advances in computational neuroscience have revealed a promising alternative rooted in the dynamics of spiking neural networks [8]. Building on seminal work connecting recurrent neural networks to optimization [9,10,11], researchers have demonstrated that leaky integrate-and-fire (LIF) neuron models naturally implement projected gradient descent for constrained convex programs [12,13,14]. In this framework, continuous membrane potential integration corresponds to gradient flow on an objective function, while discrete threshold-triggered spike events enforce constraints through boundary projections. Stanojevic et al. [15] recently showed this principle scales to deep architectures with remarkable efficiency, achieving competitive performance with only 0.3 spikes per neuron. This sparsity metric demonstrates the computational efficiency of spike-based constraint enforcement: the low spike rate indicates that SNN dynamics can achieve effective optimization with minimal discrete events, making the approach computationally efficient and suitable for real-time applications. The 0.3 spikes per neuron result, achieved on image classification tasks, illustrates that spiking networks can match artificial neural network performance while maintaining extreme sparsity in their communication patterns. For portfolio optimization, this suggests that constraint violations can be corrected efficiently through sparse projection spikes rather than continuous monitoring, enabling energy-efficient implementation on neuromorphic hardware. Readers interested in the theoretical foundations and training dynamics underlying this sparsity result are referred to the original publication [15] for detailed analysis. This biological computation model suggests an algorithmic strategy: if portfolio optimization can be reformulated as a constrained quadratic program compatible with LIF dynamics, it becomes amenable to implementation on specialized neuromorphic processors designed to execute spiking computations with extreme energy efficiency.

This work aligns with the core principles of biomimetics by drawing direct inspiration from biological neural computation mechanisms. Biological neurons achieve remarkable computational efficiency through event-driven signaling: rather than continuously transmitting information, neurons integrate inputs over time and emit discrete action potentials (spikes) only when threshold conditions are met. This sparse, asynchronous communication strategy enables biological neural networks to process complex information while maintaining extremely low energy consumption. The human brain operates on approximately 20 watts, far less than conventional computers performing similar computational tasks. Our approach mimics this biological strategy by translating portfolio optimization into a framework where continuous gradient integration (analogous to membrane potential dynamics) alternates with discrete projection spikes (analogous to action potentials) that enforce constraints. This biomimetic design not only provides algorithmic advantages but also establishes a natural pathway to neuromorphic hardware deployment, where specialized processors can execute these dynamics with the same energy-efficient, event-driven architecture that biological systems employ. By learning from nature’s computational strategies, we demonstrate how biomimetic principles can address fundamental challenges in quantitative finance while opening new avenues for energy-efficient computing.

The biomimetic perspective extends beyond mere algorithmic inspiration to encompass the broader philosophy of learning from biological systems to solve engineering challenges. Just as biomimetics has informed innovations in materials science (e.g., gecko-inspired adhesives), robotics (e.g., bird-inspired flight), and energy systems (e.g., photosynthesis-inspired solar cells), our work demonstrates how neural computation strategies can inform optimization algorithms. The key insight is that biological systems have evolved highly efficient solutions to complex problems through millions of years of natural selection, and these solutions often outperform engineered alternatives in terms of energy efficiency, robustness, and adaptability. By adopting the event-driven, sparse communication paradigm of biological neurons, we develop an optimization approach that inherits these advantages: reduced computational overhead, natural parallelization, and compatibility with emerging neuromorphic hardware platforms. This interdisciplinary synthesis of neuroscience, optimization theory, and computational finance exemplifies how biomimetic principles can drive innovation across traditional disciplinary boundaries.

This connection is more than theoretical. Neuromorphic hardware platforms such as Intel Loihi [16,17] and SpiNNaker [18] implement massively parallel, event-driven architectures optimized for spiking neural network execution. IBM’s TrueNorth [19] demonstrated that a million-neuron chip could operate with remarkable energy efficiency. Unlike traditional von Neumann processors that execute sequential instructions, these systems compute through distributed local operations, precisely the structure required for gradient-and-projection dynamics. Deploying portfolio optimization on such hardware could compress multi-second runtimes to microseconds while consuming orders of magnitude less energy, enabling real-time rebalancing at institutional scales currently impractical for conventional solvers.

This paper develops the mathematical and algorithmic foundation for neuromorphic portfolio optimization. We reformulate the transaction-cost-aware, cardinality-constrained Markowitz model as a constrained quadratic program with linear inequalities, implement an SNN-inspired solver that alternates between continuous gradient integration and discrete projection spikes, and demonstrate through comprehensive experiments on real equity data that the approach produces solutions competitive with exact mixed-integer programming and superior to metaheuristics. While our Python proof-of-concept achieves runtimes comparable to conventional methods, the primary contribution extends beyond current performance: we establish the pathway to neuromorphic deployment that could revolutionize computational finance through hardware-accelerated optimization.

Specifically, the main contributions are as follows:

1. Theoretical formulation: We establish the mathematical connection between constrained portfolio optimization and LIF neuron dynamics, reformulating the mixed-integer Markowitz model as a constrained quadratic program where gradient descent drives portfolio weights toward optimality while projection spikes enforce relaxed continuous constraints during optimization (including transaction cost budgets and relaxed binary selectors), with discrete cardinality requirements recovered through deterministic post-processing.
2. Algorithm realization: We implement the gradient-and-spike dynamics as a practical solver (Section 4) using adaptive ODE integration with event detection for constraint violations, demonstrating that the alternating continuous-discrete structure achieves $O(N^{2})$ per-step complexity (where “per-step” refers to one ODE integration step during the continuous gradient descent phase) dominated by covariance matrix operations, with projection spikes requiring only O(N) operations due to sparse matrix-vector products. These operations are naturally parallelizable on neuromorphic hardware.
3. Comprehensive evaluation: We conduct systematic parameter sweeps across four portfolio sizes using five years of prices for 100 liquid equities, compare against exact MIQP (ECOS_BB), convex relaxation (OSQP + $\ell_{1}$), and particle swarm optimization, and demonstrate that the SNN approach lies on the Pareto frontier of runtime versus solution quality while uniquely enabling neuromorphic deployment.
4. Hardware deployment pathway: We analyze the computational characteristics that make the SNN dynamics amenable to specialized processors, describe the mapping from algorithm to neuromorphic architecture, and quantify the potential speedup from event-driven parallel execution on platforms like Intel Loihi.

The remainder of the paper proceeds as follows. Section 2 positions our work relative to neural dynamics for optimization, neuromorphic computing, and portfolio selection methods. Section 3 establishes the mathematical foundation connecting LIF dynamics to projected gradient descent and formalizes the constrained portfolio problem. Section 4 describes the solver implementation and discusses neuromorphic hardware considerations. Section 5 presents experimental validation across multiple scales and baseline comparisons. Section 6 concludes with limitations and future directions for hardware deployment.

## 2. Related Work

This section positions our contribution relative to three interconnected research areas: the theoretical foundations connecting neural dynamics to constrained optimization, the evolution of neuromorphic computing platforms that enable efficient implementation of spiking networks, and the portfolio optimization literature that has explored both exact and heuristic methods for handling transaction costs and cardinality constraints. We emphasize how our work synthesizes insights from these domains to establish a practical pathway for hardware-accelerated financial optimization.

### 2.1. Neural Dynamics for Constrained Optimization

The connection between neural networks and optimization has deep roots in computational neuroscience. Hopfield [9,10] pioneered this area by demonstrating that recurrent networks with symmetric connections perform gradient descent on energy functions, establishing that neural dynamics can solve optimization problems through collective computational processes. This seminal work showed that biological neural circuits naturally minimize objectives through distributed local operations; a principle that remains central to modern neuromorphic computing.

Building on Hopfield’s foundation, researchers developed projection neural networks specifically designed for constrained optimization [20,21]. These networks implement projected gradient descent through continuous-time dynamics that alternate between gradient flow and boundary projections, directly enforcing linear inequality constraints. Xia and Wang [21] established global convergence guarantees for projection networks solving monotone variational inequalities, demonstrating that neural architectures can reliably solve constrained convex programs. Liu and Wang [22] extended this framework to handle both equality constraints and box constraints simultaneously, showing that single-layer networks suffice for complex feasible regions when equipped with appropriate projection operators.

Recent advances in spiking neural networks have strengthened the optimization connection by linking discrete spike events to constraint enforcement. Boerlin et al. [13] showed that networks of spiking neurons perform predictive coding through a form of constrained optimization, where spike events minimize prediction errors subject to metabolic costs. Barrett and Deneve [12] proved that networks of leaky integrate-and-fire neurons implicitly solve convex optimization problems, where membrane voltage thresholds implement inequality constraints and spike timing encodes optimal solutions. This work revealed that the alternation between continuous integration and discrete resets, the hallmark of LIF dynamics, directly corresponds to projected gradient descent. Mancoo et al. [14] unified these insights by demonstrating that balanced spiking networks solve quadratic programs with linear constraints, establishing the mathematical equivalence between spike-based computation and convex optimization that forms the theoretical foundation of our work. Stanojevic et al. [15] demonstrated that this principle scales to deep architectures, achieving competitive performance on recognition tasks while maintaining the sparse, event-driven computation that makes SNNs amenable to neuromorphic hardware. Recent developments in SNN training methodologies have further advanced the field: Perin et al. [23] introduced an alternating direction method of multipliers (ADMM) approach for SNN training that addresses the non-differentiability of spike functions while providing convergence guarantees, demonstrating that principled optimization frameworks can enhance SNN performance and reliability.

Our work extends this theoretical lineage to financial optimization. While previous projection networks focused on general constrained programs or robotic control [24], we specialize the framework to portfolio selection with transaction costs and cardinality requirements. Crucially, we identify the specific problem structure (constrained quadratic program with sparse linear inequalities) that enables mapping to LIF dynamics, and demonstrate that the resulting solver achieves solution quality competitive with exact mixed-integer methods while uniquely enabling neuromorphic deployment. Table 1 summarizes this theoretical progression from Hopfield’s pioneering work to modern SNN-based optimization, positioning our contribution within this intellectual lineage.

### 2.2. Neuromorphic Computing Platforms

The algorithmic insights linking neural dynamics to optimization would remain theoretical without specialized hardware capable of efficient SNN execution. Neuromorphic processors address this need through radically different architectures than traditional von Neumann machines. IBM’s TrueNorth [19] pioneered large-scale neuromorphic integration with a million-neuron chip operating at ultra-low power, demonstrating that spike-based computation could be implemented efficiently in silicon. Intel’s Loihi [16] implements asynchronous, event-driven computation where individual neurons communicate through discrete spike events, consuming energy only when active. This matches the sparse communication patterns of biological neural networks and the projection-spike structure of our portfolio solver. Davies et al. demonstrated that Loihi achieves 1000× energy efficiency improvements over GPUs for certain SNN workloads, validating the potential for orders-of-magnitude acceleration. Recent work [17] has further improved Loihi 2’s spike processing throughput and demonstrated efficient implementation of neuromorphic signal processing algorithms. Recent evaluations [25] have comprehensively benchmarked neuromorphic processors including Intel Loihi and BrainScaleS against standard AI accelerators across diverse application domains, demonstrating competitive performance with substantially lower power consumption for spike-based algorithms.

SpiNNaker [18] takes a complementary approach, implementing massively parallel ARM cores specialized for spike routing and synaptic processing. The architecture supports millions of simulated neurons executing in real time, enabling large-scale network deployment. Recent extensions have improved spike processing throughput and on-chip learning capabilities, making these platforms increasingly viable for practical applications beyond neuroscience research. Contemporary research [26] has addressed the practical challenges of deploying SNNs on neuromorphic hardware by developing training methods that explicitly account for hardware constraints such as limited neuron and synapse counts and low bit-width representations, enabling efficient real-world deployment of SNN-based algorithms.

The key architectural feature enabling our portfolio optimization application is the natural mapping between algorithm primitives and neuromorphic operations. Gradient descent (continuous voltage integration) maps to leak dynamics inherent in LIF neurons. Constraint checking (inequality evaluation) corresponds to threshold comparison. Projection spikes (discrete corrections) align with neuronal reset events. Matrix-vector products for covariance evaluation distribute across parallel synaptic operations. This structural compatibility distinguishes our SNN-based approach from both exact solvers, which require sequential branching operations unsuitable for parallel hardware, and metaheuristics, which lack a principled mapping to specialized processors. Table 2 summarizes the capabilities of major neuromorphic platforms and their suitability for deploying optimization algorithms like ours.

### 2.3. Portfolio Optimization Methods

The portfolio optimization literature has explored diverse approaches to handle transaction costs and cardinality constraints, each with characteristic trade-offs. Salo et al. [28] provide a comprehensive review of fifty years of portfolio optimization research, documenting the evolution from classical mean-variance theory to modern approaches incorporating machine learning, robust optimization, and alternative risk measures, highlighting the persistent computational challenges posed by real-world constraints. Exact methods based on mixed-integer programming [3,4] formulate the problem with binary variables indicating asset selection and apply branch-and-bound algorithms to explore the combinatorial solution space. While provably optimal, these methods exhibit exponential worst-case complexity, making them impractical for large universes or time-critical applications. Extensions incorporating robust optimization [29,30] or risk measures beyond variance [31] further increase computational burden, motivating research on faster alternatives.

Metaheuristic algorithms offer computational tractability by replacing exhaustive search with stochastic exploration [32,33]. Particle swarm optimization [6,34] maintains populations of candidate portfolios that evolve based on individual and collective performance. Genetic algorithms [35,36] encode portfolios as binary strings and apply selection, crossover, and mutation operators inspired by biological evolution. Beetle antennae search [37] reduces population requirements through targeted sampling. Khan et al. [7] provided a comprehensive review of metaheuristic approaches for cardinality-constrained portfolios, highlighting their computational advantages but noting the lack of optimality guarantees. While these methods find acceptable solutions more quickly than exact approaches, they require extensive parameter tuning, lack theoretical convergence analysis, and provide no clear path to hardware acceleration.

Recent work has integrated machine learning into portfolio selection, using deep neural networks for return prediction [38] or reinforcement learning for dynamic rebalancing [39]. However, these sophisticated learning approaches increase rather than decrease computational requirements, making real-time deployment challenging. They also do not address the fundamental mixed-integer structure of cardinality constraints.

Modern convex optimization solvers [40,41] provide efficient solutions for continuous relaxations of portfolio problems. OSQP (Operator Splitting Quadratic Program) [40] achieves remarkable performance for large-scale quadratic programs through first-order splitting methods, making it a strong baseline for comparison. However, these solvers require post-processing to recover discrete solutions and lack the hardware acceleration pathway offered by neuromorphic computing.

Early research on transaction costs [2] established that even small trading fees significantly alter optimal portfolios, leading to sparse solutions where many assets receive zero allocation. This observation motivates the cardinality constraint formulation we adopt: explicitly limiting portfolio size through integer constraints rather than relying on regularization to induce sparsity. Our SNN-based approach addresses the resulting mixed-integer program through continuous relaxation during optimization combined with deterministic post-processing for discrete recovery, achieving a practical middle ground between exact methods and heuristics.

Critically, none of the existing portfolio optimization methods, exact, metaheuristic, or machine learning-based, offer a clear pathway to specialized hardware acceleration. Exact solvers require sequential decision trees incompatible with parallel architectures. Metaheuristics involve population management and random sampling that map poorly to neuromorphic substrates. Learning-based methods require substantial training infrastructure. Our SNN formulation uniquely bridges computational finance and neuromorphic computing by establishing the theoretical connection (constrained QP to LIF dynamics), implementing a practical solver (gradient-and-spike algorithm), and demonstrating competitive solution quality that justifies the hardware investment.

### 2.4. Comparison with Existing Optimization Algorithms

To clarify the distinctive characteristics of our SNN-based approach, we explicitly contrast it with existing optimization methods across several key dimensions: algorithmic structure, computational complexity, constraint handling, and hardware deployment potential.

SNN vs. Exact MIQP Solvers: Exact mixed-integer quadratic programming methods [3,4] such as ECOS_BB employ branch-and-bound algorithms that systematically explore the discrete solution space through sequential decision trees. While these methods provide optimality guarantees, they exhibit exponential worst-case complexity and require sequential branching operations that cannot be parallelized effectively. In contrast, our SNN approach solves a relaxed continuous problem using parallel gradient-projection dynamics, recovering discrete solutions through deterministic post-processing. This relaxation-then-recovery strategy allows exploration of a broader solution space before discrete constraint enforcement, potentially identifying superior local optima as demonstrated in our experimental results (Section 5). Critically, the SNN dynamics map naturally to parallel neuromorphic hardware [16,17], while branch-and-bound algorithms fundamentally require sequential execution incompatible with specialized processors.

SNN vs. Convex Relaxation Methods: Convex relaxation approaches [40,41] such as OSQP + $\ell_{1}$ solve continuous approximations efficiently but require post-processing heuristics to recover discrete solutions. These methods lack principled mechanisms for enforcing cardinality constraints during optimization, relying instead on regularization [42] to promote sparsity followed by thresholding. Our SNN method integrates constraint enforcement directly into the optimization dynamics through projection spikes, providing a principled mechanism for maintaining feasibility throughout the solution process. While both approaches use post-processing for final discrete recovery, the SNN dynamics naturally drive selector variables toward binary values (0 or 1) through the optimization trajectory, making the recovery procedure more reliable [43,44]. Additionally, the SNN formulation provides a clear pathway to neuromorphic hardware deployment [25,26], while convex solvers remain limited to conventional architectures.

SNN vs. Metaheuristic Approaches: Metaheuristic methods such as particle swarm optimization [5,6] and genetic algorithms [36] replace exhaustive search with stochastic exploration, trading optimality guarantees for computational speed. These methods require extensive parameter tuning, lack theoretical convergence analysis, and involve population management and random sampling operations that map poorly to specialized hardware. Khan et al. [7] provided a comprehensive review of metaheuristic approaches for cardinality-constrained portfolios, highlighting their computational advantages but noting the lack of optimality guarantees. Our SNN approach builds on the theoretical foundation of projected gradient descent [12,21] for constrained quadratic programs, providing principled convergence behavior and requiring minimal parameter tuning (two key parameters $k_{0}$ and $k_{1}$ with clear physical interpretations). The deterministic gradient-projection structure maps directly to neuromorphic hardware, while metaheuristics’ stochastic nature precludes efficient hardware acceleration.

SNN vs. Other Neural Dynamics Methods: Previous projection neural networks [20,21] implement continuous-time dynamics for constrained optimization but operate in analog voltage space without discrete spike events. While these methods share the projected gradient descent foundation, they lack the event-driven computation model that enables neuromorphic hardware deployment. Our SNN approach explicitly models discrete spike events that correspond to constraint violations, mirroring the biological computation model and enabling direct mapping to spike-based neuromorphic processors. This spike-based structure provides natural sparsity (most neurons remain silent), enabling energy-efficient implementation on specialized hardware designed for event-driven computation.

The unique advantage of our SNN formulation lies in its dual nature: it combines the theoretical rigor of projected gradient descent with the practical pathway to neuromorphic hardware deployment. Unlike exact solvers (sequential branching), convex relaxations (no hardware mapping), or metaheuristics (stochastic operations), the SNN dynamics operate through simple local computations: vector operations, threshold comparisons, and fixed-magnitude updates that map directly to neuromorphic processor architectures. This structural compatibility, combined with competitive solution quality demonstrated in our experiments, establishes the SNN approach as a promising direction for real-time portfolio optimization at institutional scales.

## 3. Preliminaries

This section establishes the mathematical foundation for our approach. We first review the leaky integrate-and-fire neuron model and explain how its dynamics naturally implement projected gradient descent for constrained optimization. We then formulate the portfolio selection problem with transaction costs and cardinality constraints, showing how it can be cast as a constrained quadratic program amenable to SNN dynamics.

### 3.1. Spiking Neural Network Dynamics

Spiking neural networks model individual neurons as dynamic systems that integrate input currents over time and emit discrete spike events when an internal state variable crosses a threshold. The leaky integrate-and-fire (LIF) neuron, the most widely studied SNN model, captures essential computational properties while remaining mathematically tractable.

#### 3.1.1. Leaky Integrate and Fire Neuron Model

The membrane potential dynamics of a single LIF neuron follow the first-order differential equation

(1) $$\tau_{m}\frac{dV_{m}}{dt}=-\left(V_{m}-V_{\mathrm{rest}}\right)+R_{m}I\left(t\right)$$

where $V_{m}\left(t\right)$ represents the membrane potential, $\tau_{m}$ is the membrane time constant, $V_{\mathrm{rest}}$ denotes the resting potential, $R_{m}$ is the membrane resistance, and I(t) represents the input current.

The dynamics in (1) describe a leaky integrator: when I(t)=0, the membrane potential exponentially decays toward $V_{\mathrm{rest}}$ with time constant $\tau_{m}$. Positive input currents drive $V_{m}$ above $V_{\mathrm{rest}}$, while negative currents drive it below.

When the membrane potential reaches a threshold value $V_{\mathrm{th}}$, the neuron emits a spike and the voltage undergoes a discrete reset:

(2) $$\mathrm{if}\;V_{m}\geq V_{\mathrm{th}},\;\mathrm{then}\;V_{m}\leftarrow V_{\mathrm{reset}}$$

where $V_{\mathrm{reset}}<V_{\mathrm{th}}$ is the reset potential. For our optimization application, we focus on these essential threshold and reset mechanics; secondary physiological details such as refractory periods are not required.

#### 3.1.2. Connection to Constrained Optimization

The key insight connecting LIF dynamics to optimization is recognizing that voltage thresholds naturally implement inequality constraints. Consider a constrained optimization problem

(3) $$\begin{array}{cc} \underset{y}{\mathrm{minimize}}\; & f(y)\\ \mathrm{subject}\;\mathrm{to}\; & Cy+d\leq 0 \end{array}$$

where $y\in R^{n}$ is the optimization variable, $f:R^{n}\to R$ is the objective function, $C\in R^{m\times n}$ defines *m* linear inequality constraints, and $d\in R^{m}$ specifies constraint offsets.

Gradient descent on the objective follows

(4) $$\frac{dy}{dt}=-k_{0}\nabla f\left(y\right)$$

where $k_{0}>0$ is the step size and ∇f denotes the gradient. Comparing (4) with the LIF dynamics (1), we observe a structural correspondence: if we interpret $V_{m}$ as y and I(t) as encoding negative gradient information, the neuron dynamics implement gradient descent.

The threshold mechanism (2) in LIF neurons provides a natural way to enforce constraints. When constraint violations are detected (analogous to $V_{m}\geq V_{\mathrm{th}}$), a discrete correction event (spike) projects the state back toward feasibility. If a set of constraints V is violated (i.e., rows with ${\left[Cy+d\right]}_{i}>0$), we apply a correction along their signed normals:

(5) $$y\leftarrow y-k_{1}\;C^{⊤}1_{V}$$

where $k_{1}>0$ controls the spike magnitude and $1_{V}\in R^{m}$ is an indicator vector with ${\left[1_{V}\right]}_{i}=1$ if constraint *i* is violated (i.e., ${\left[Cy+d\right]}_{i}>0$) and ${\left[1_{V}\right]}_{i}=0$ otherwise, where $V=\{i:{\left[Cy+d\right]}_{i}>0\}$ denotes the set of violated constraint indices. These repeated corrections resemble LIF resets and keep the trajectory close to the feasible region.

This alternation between continuous gradient descent (4) and discrete constraint projections (5) forms the basis of our SNN-inspired optimization algorithm. The continuous phase minimizes the objective, while discrete correction events maintain feasibility, analogous to how neurons integrate inputs continuously but generate spikes discretely when thresholds are crossed.

### 3.2. Portfolio Optimization Problem Formulation

We now formulate the constrained portfolio optimization problem that will be solved using the SNN-inspired framework in Section 4. The formulation extends the classical Markowitz mean-variance model with transaction costs and cardinality constraints to reflect realistic trading conditions.

#### 3.2.1. Basic Notation and Markowitz Model

Consider a financial market with *N* available stocks denoted $S_{1},S_{2},\ldots ,S_{N}$. Historical price data is used to estimate the mean return rate $\mu_{i}$ and return covariance $\sigma_{ij}$ for each pair of stocks, where i,j∈{1,2,…,N}. We organize these statistics into a mean return vector $\mu \in R^{1\times N}$ and covariance matrix $\Sigma \in R^{N\times N}$:

(6) $$\begin{array}{cc} \mu & =\left[\begin{array}{cccc} \mu_{1} & \mu_{2} & \ldots & \mu_{N} \end{array}\right] \end{array}$$

(7) $$\begin{array}{cc} \Sigma & =\left[\begin{array}{cccc} \sigma_{11} & \sigma_{12} & \ldots & \sigma_{1N}\\ \sigma_{21} & \sigma_{22} & \ldots & \sigma_{2N}\\ \vdots & \vdots & \ddots & \vdots \\ \sigma_{N1} & \sigma_{N2} & \ldots & \sigma_{NN} \end{array}\right] \end{array}$$

The covariance matrix Σ is symmetric with $\sigma_{ij}=\sigma_{ji}$, and positive semidefinite under standard assumptions.

Let $t=[t_{1},t_{2},\ldots ,t_{N}]$ denote the portfolio allocation vector, where $t_{i}$ represents the fraction of total capital invested in stock $S_{i}$. We normalize the total investment to unity, so $\sum_{i=1}^{N}t_{i}=1$ or equivalently $1t^{⊤}=1$ where 1 is a row vector of ones.

The expected return of the portfolio is given by

(8) $$\bar{\mu }\left(t\right)=t\mu^{⊤}$$

and the portfolio variance, representing risk, is

(9) $$\bar{\Sigma }\left(t\right)=t\Sigma t^{⊤}$$

The classical Markowitz mean-variance optimization seeks to minimize risk for a target expected return $\mu_{req}$:

(10) $$\begin{array}{cc} \underset{t}{\mathrm{minimize}}\; & t\Sigma t^{⊤}\\ \mathrm{subject}\;\mathrm{to}\; & t\mu^{⊤}=\mu_{req}\\ \; & 1t^{⊤}=1\\ \; & 0\leq t_{i}\leq 1,\;i=1,\ldots ,N \end{array}$$

While this formulation is elegant and admits efficient solution methods, it lacks several features necessary for practical implementation.

#### 3.2.2. Transaction Costs

Real-world trading incurs transaction costs including brokerage fees, bid-ask spreads, and market impact. A common model assumes costs proportional to trade size, leading to the linear transaction cost model. Let $\alpha_{i}$ denote the transaction cost rate for stock $S_{i}$, organized into a vector $\alpha =[\alpha_{1},\alpha_{2},\ldots ,\alpha_{N}]$. In our experiments the entries of α are drawn uniformly and scaled so that $\sum_{i}\alpha_{i}=0.1$, yielding an average 10% surcharge across the universe. The total cost for establishing portfolio t is

(11) $$\mathrm{Transaction}\;\mathrm{Cost}=\alpha t^{⊤}$$

Since the investor must pay both for the shares and the transaction costs, the budget constraint becomes

(12) $$\left(1+\alpha \right)t^{⊤}=1$$

This replaces the simple budget constraint $1t^{⊤}=1$ in the frictionless Markowitz model.

#### 3.2.3. Cardinality Constraint

Investors often wish to limit the number of stocks held in a portfolio to reduce monitoring costs, improve interpretability, and comply with regulatory requirements. The cardinality constraint specifies that at most *k* stocks may receive nonzero allocation, where k<N.

To formulate this constraint, we introduce binary decision variables $z=[z_{1},z_{2},\ldots ,z_{N}]$ where $z_{i}=1$ if stock $S_{i}$ is included in the portfolio and $z_{i}=0$ otherwise. The cardinality constraint is then

(13) $$\sum_{i=1}^{N}z_{i}=k\;\mathrm{or}\;\mathrm{equivalently}\;1z^{⊤}=k$$

We must also ensure that stocks excluded from the portfolio receive zero allocation, which is enforced by coupling the continuous allocation variables t with the binary selection variables z through

(14) $$0\leq t_{i}\leq z_{i},\;i=1,2,\ldots ,N$$

When $z_{i}=0$, this forces $t_{i}=0$, while $z_{i}=1$ allows $t_{i}\in \left[0,1\right]$.

#### 3.2.4. Unified Optimization Formulation

We adopt an objective function that incorporates both risk minimization and return maximization rather than fixing a target return. Specifically, we minimize a weighted combination of variance and negative return:

(15) $$E\left(t\right)=t\Sigma t^{⊤}-\Lambda t\mu^{⊤}$$

where the parameter Λ≥0 controls the risk-return tradeoff. Larger Λ places more emphasis on maximizing returns, while smaller values prioritize risk reduction. The negative sign on the return term ensures that minimizing *E* corresponds to maximizing returns.

Combining the objective (15) with all constraints, the complete portfolio optimization problem is

(16) $$\begin{array}{cc} \underset{t,z}{\mathrm{minimize}}\; & t\Sigma t^{⊤}-\Lambda t\mu^{⊤}\\ \mathrm{subject}\;\mathrm{to}\; & \left(1+\alpha \right)t^{⊤}=1\\ \; & 0\leq t_{i}\leq z_{i},\;i=1,\ldots ,N\\ \; & 1z^{⊤}=k\\ \; & z\in {\left\{0,1\right\}}^{1\times N} \end{array}$$

where the minimization is performed over both the continuous portfolio weights t and the binary selection variables z. The objective function depends on t through the quadratic risk term $t\Sigma t^{⊤}$ and the linear return term $t\mu^{⊤}$, while z enters only through the constraints.

This formulation presents significant computational challenges. The binary variables z make it a mixed-integer nonlinear program, which is NP-hard in general. Traditional methods such as branch-and-bound become expensive as *N* grows, motivating alternative approaches.

The key to enabling SNN-based solution is reformulating this mixed-integer problem as a constrained quadratic program. In Section 4, we relax the binary constraint $z\in {\left\{0,1\right\}}^{1\times N}$ to a continuous box $z\in {\left[0,1\right]}^{1\times N}$ during optimization, express all constraints as linear inequalities compatible with the projection mechanism (5), and leverage SNN dynamics to maintain feasibility. After the dynamics settle, a deterministic post-processing step reinstates discrete portfolios satisfying the cardinality limit, as described in Section 4.

## 4. SNN-Inspired Optimization Method

This section describes our implementation of the SNN-inspired solver for portfolio optimization. We first show how the mixed-integer Markowitz model from Section 3 is reformulated as a constrained quadratic program amenable to SNN dynamics. We then describe the solver algorithm that alternates between continuous gradient integration and discrete projection spikes, detail the post-processing procedure that recovers discrete portfolios, and discuss the computational characteristics that make the approach suitable for neuromorphic hardware deployment.

### 4.1. Quadratic Program Reformulation

To enable SNN-based solution, we reformulate the mixed-integer problem (16) as a constrained quadratic program. The solver accepts problems of the form

(17) $$\underset{y}{\mathrm{minimize}}\;\frac{1}{2}y^{⊤}Ay+b^{⊤}y\;\mathrm{subject}\;\mathrm{to}\;Cy+d\leq 0,$$

where $y\in R^{2N}$ contains both portfolio weights and relaxed binary selectors stacked as

(18) $$y={\left[\begin{array}{cc} t^{⊤} & z^{⊤} \end{array}\right]}^{⊤}.$$

The binary constraint $z\in {\left\{0,1\right\}}^{N}$ is relaxed to $z\in {\left[0,1\right]}^{N}$ during optimization; discrete portfolios are recovered through post-processing (Section 4).

The Hessian and linear term encode the Markowitz objective (15) with risk-return trade-off parameter Λ:

(19) $$A=\left[\begin{array}{cc} 2\Sigma & 0\\ 0 & 0 \end{array}\right],\;b=\left[\begin{array}{c} -\Lambda \mu^{⊤}\\ 0 \end{array}\right].$$

The block structure ensures that only portfolio weights t appear in the quadratic term (through Σ), while relaxed selectors z enter only via constraints.

All constraints from (16) are expressed as linear inequalities compatible with the projection mechanism (5). The budget constraint $\left(1+\alpha \right)t^{⊤}=1$ becomes two inequalities with tolerance $\epsilon_{\mathrm{budget}}$; box constraints $0\leq t_{i}\leq z_{i}$ and $0\leq z_{i}\leq 1$ contribute 4N rows; and the cardinality requirement $1z^{⊤}=k$ is enforced with upper and lower bounds using tolerance $\epsilon_{\mathrm{card}}$. The resulting constraint matrix $C\in R^{(4N+4)\times 2N}$ is sparse and problem-specific, so we precompute it once per scenario along with offsets d. This allows rapid re-instantiation for different $\left(k_{0},k_{1}\right)$ parameter sweeps.

### 4.2. SNN Dynamics and Projection Algorithm

The core solver implements the alternating gradient-and-spike dynamics described in Section 3. Algorithm 1 summarizes the main loop. The configuration is specified by parameters $\left(k_{0},k_{1},t_{\max},h_{\max},\epsilon_{\mathrm{tol}}\right)$, where $k_{0}$ controls the gradient descent rate, $k_{1}$ sets the projection spike magnitude, $t_{\max}$ is the simulation time horizon, $h_{\max}$ caps the ODE integration step size, and $\epsilon_{\mathrm{tol}}$ is the constraint satisfaction tolerance.

Between projection events, the state evolves according to the gradient flow

(20) $$\frac{dy}{dt}=-k_{0}\;\left(Ay+b\right).$$

Due to the block structure (19), this updates only the portfolio weights t while leaving relaxed selectors z unchanged during continuous descent. We integrate using an adaptive Runge–Kutta RK45 scheme with event detection to halt when constraint violations are imminent. The maximum step size $h_{\max}=0.1$ ensures smooth trajectories and simplifies spike time reconstruction.

Each integration step produces a trajectory segment $\left(t_{\mathrm{seg}},y_{\mathrm{seg}}\right)$ containing the time points and state values along the continuous gradient descent path. The segment terminates either when a constraint violation is detected (triggering a return to the projection phase) or when the integration step completes successfully. The algorithm appends each segment to the full solution trajectory and updates the current state to the final point of the segment: $t\leftarrow t_{\mathrm{seg}}\left[-1\right]$ and $y\leftarrow y_{\mathrm{seg}}\left[-1\right]$, where the [−1] notation denotes the last element of the segment arrays. This piecewise trajectory construction allows the solver to maintain a complete record of the optimization path while handling the alternating continuous-discrete dynamics efficiently.

When the event detector identifies that $\max_{i}{\left[Cy+d\right]}_{i}$ approaches zero (within $\epsilon_{\mathrm{tol}}$), the solver applies a discrete projection spike:

(21) $$y\leftarrow y-k_{1}\;C^{⊤}1_{V},$$

where $1_{V}$ selects the currently violated constraint rows. Unlike analytical projection onto the constraint boundary, this correction moves along signed constraint normals with fixed magnitude $k_{1}$, directly mirroring the reset behavior of leaky integrate-and-fire neurons. The projection is iterated (up to $N_{\max}=100$ iterations) until all constraints satisfy $Cy+d\leq \epsilon_{\mathrm{tol}}$. We record spike metadata (timestamps, active constraints, displacement norms) for diagnostic analysis in Section 5.

Integration terminates when either simulated time reaches $t_{\max}$ or the gradient norm falls below $10^{-8}$. Solutions that terminate exactly at $t_{\max}$ are flagged as time-horizon limited, indicating that longer simulation might yield further improvement.
**Algorithm 1** SNN-inspired portfolio optimization algorithm**Input:** Problem matrices (A,b,C,d), solver parameters $\left(k_{0},k_{1},t_{\max},h_{\max},\epsilon_{\mathrm{tol}}\right)$, feasible initial state $x_{0}$  1:t←0, $x\leftarrow x_{0}$  2:**while** $t<t_{\max}$ and gradient norm $>10^{-8}$ **do**  3:      $x\leftarrow \mathrm{ProjectToFeasible}(x,k_{1},\epsilon_{\mathrm{tol}})$                                          ▹Apply (21) until feasible  4:      Integrate $\dot{x}=-k_{0}\left(Ax+b\right)$ with event detection for constraint violations  5:      Append $\left(t_{\mathrm{seg}},x_{\mathrm{seg}}\right)$ to solution trajectory  6:      $t\leftarrow t_{\mathrm{seg}}\left[-1\right]$, $x\leftarrow x_{\mathrm{seg}}\left[-1\right]$  7:      **if** trajectory stalled at boundary **then**  8:            **break**  9:      **end if**10:**end while**11:**return** trajectory, spike metadata, final solution *x*

**Figure 1:**
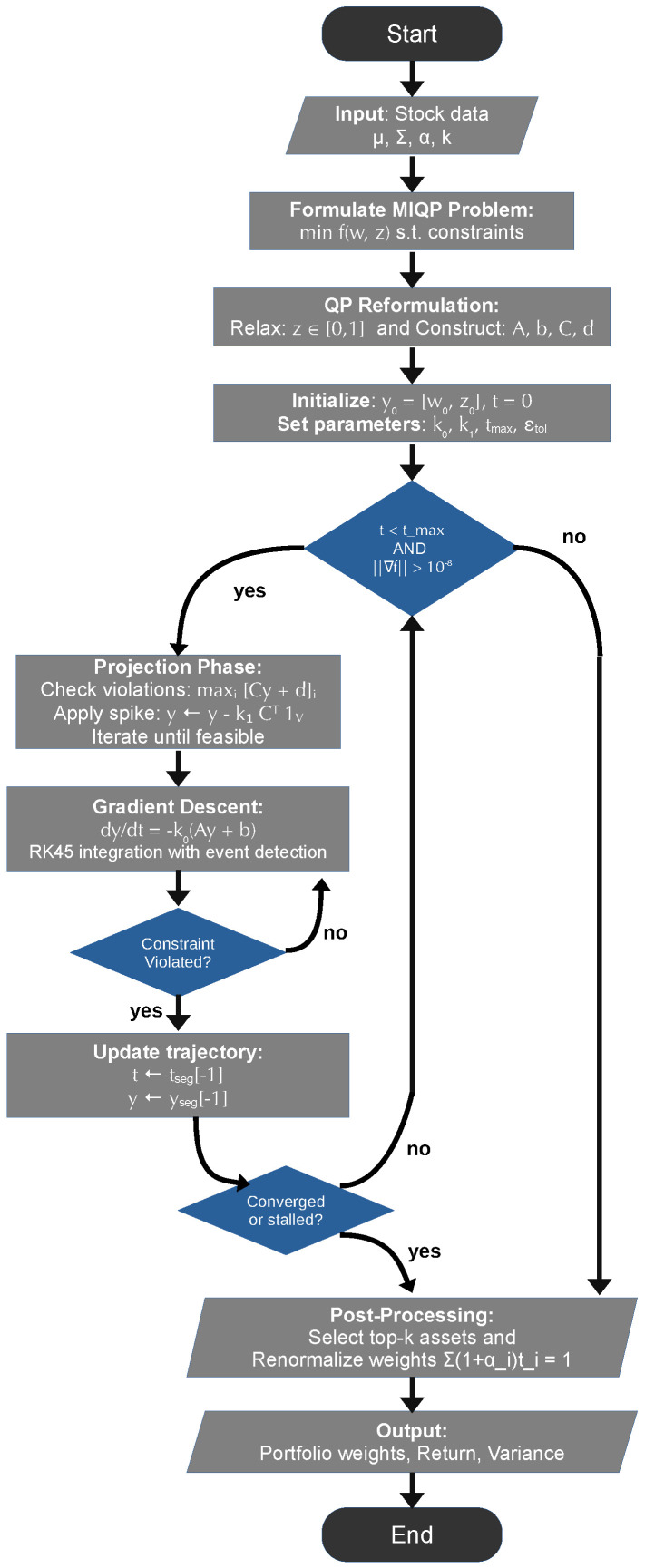
Complete workflow of the SNN-inspired portfolio optimization algorithm. The flowchart illustrates the alternating structure between continuous gradient descent (smooth trajectories) and discrete projection spikes (constraint corrections), event-driven constraint violation detection, and the deterministic post-processing step that recovers discrete portfolios from relaxed solutions.

Figure 1 provides a visual overview of the complete SNN optimization workflow, illustrating the key algorithmic components and their interactions. The flowchart highlights the alternating structure between continuous gradient descent phases and discrete projection spike events, emphasizing the event-driven nature of constraint enforcement. The main optimization loop continues until convergence criteria are met, with trajectory segments recorded throughout the process. The final post-processing step deterministically recovers a discrete portfolio satisfying the cardinality constraint, completing the transformation from relaxed continuous solution to implementable discrete allocation. This visual representation complements Algorithm 1 by showing the dynamic flow and decision points that govern the solver’s behavior.

### 4.3. Discrete Portfolio Recovery and Validation

After the SNN dynamics converge, we recover a discrete portfolio that satisfies the original cardinality constraint $1z^{⊤}=k$. The relaxed selector values z produced by the solver are treated as continuous scores indicating asset importance. We select the *k* assets with the largest $z_{i}$ values, zero out weights for unselected assets, clip the remaining weights to [0,∞), and renormalize to satisfy the budget constraint with transaction costs:

(22) $$\sum_{i\in S}\left(1+\alpha_{i}\right)t_{i}=1,$$

where S denotes the set of selected assets. This deterministic top-*k* recovery procedure is applied uniformly across all experiments.

The binary relaxation strategy is theoretically justified by convex relaxation theory for cardinality-constrained optimization [43,44]. When the relaxed selectors z exhibit strong bimodality (many values near 0 or 1), the continuous relaxation provides a good approximation to the discrete problem, and the top-*k* recovery procedure reliably identifies the optimal discrete solution. This occurs because the optimization naturally drives selector values toward the boundaries, with assets that contribute strongly to the objective receiving values near 1, while less important assets converge toward 0. The top-*k* selection based on relaxed selector magnitudes effectively captures this importance ranking. This approach is well-established in sparse optimization literature [42], where convex relaxations of cardinality constraints (such as $\ell_{1}$ regularization) followed by thresholding provide near-optimal solutions when the solution exhibits sparsity structure. The relaxation strategy may perform less well when many selector values cluster near 0.5, indicating ambiguity in asset selection, but empirical results demonstrate that SNN dynamics naturally promote bimodal distributions, making the recovery procedure effective across all tested scenarios.

Post-processing constraint residuals are computed and recorded alongside objective value, expected return, variance, and spike statistics in the experimental summaries. This validation ensures consistency across both parameter sweeps and baseline comparisons.

### 4.4. Parameter Configuration and Tolerance Settings

Section 5 explores solver configurations $\left(k_{0},k_{1},t_{\max}\right)$ across four portfolio sizes. Conservative schedules such as $\left(k_{0},k_{1}\right)=\left(0.02,0.05\right)$ prioritize stability for small portfolios (N∈{5,10}), while more aggressive settings like (0.007,0.75) are explored for the largest case (N=50).

Constraint tolerance settings are adapted to problem size. For small portfolios (N<20), we use tight tolerances $\left(\epsilon_{\mathrm{budget}},\epsilon_{\mathrm{card}}\right)=\left(10^{-6},10^{-6}\right)$ to enforce near-exact constraint satisfaction. For larger portfolios (N≥20), these are relaxed to (0.2,0.5) to accommodate more aggressive projection gains $k_{1}$ while maintaining numerical stability. The projection tolerance $\epsilon_{\mathrm{tol}}$ is fixed at $10^{-10}$ throughout, and the maximum integration step size $h_{\max}=0.1$ for all experiments.

### 4.5. Computational Characteristics and Hardware Deployment

The solver’s computational profile makes it well-suited for neuromorphic hardware deployment. Each projection spike requires a sparse matrix-vector multiplication with $C^{⊤}$ costing O(N) operations due to the block structure, while gradient evaluations cost $O(N^{2})$ due to the dense covariance matrix Σ. Empirically, we observe fewer than three projection iterations per spike event and fewer than $2\times 10^{5}$ gradient evaluations even for the largest portfolios (N=50), keeping total solve times within single-digit seconds on conventional CPUs.

The algorithmic primitives: vector-matrix products, element-wise comparisons, and fixed-magnitude updates, map directly to the computational model of neuromorphic processors. Unlike traditional optimization methods requiring matrix factorizations or complex data structures, the SNN dynamics operate through simple local computations that can be massively parallelize. Specialized hardware such as Intel Loihi [16] or SpiNNaker2 [18] implements these operations in event-driven, low-power circuits designed specifically for spiking neural network execution.

Our Python implementation serves as a proof of concept demonstrating solution quality and algorithmic feasibility. It is important to note that neuromorphic computing represents an emerging technology, and the primary contribution of this work lies in developing an SNN-friendly problem formulation and algorithm that maps naturally to spiking neural network dynamics. Our current implementation simulates the SNN mechanics on conventional computing hardware (standard CPUs) to validate the algorithmic approach and demonstrate competitive solution quality. While the algorithmic primitives: vector operations, threshold comparisons, and fixed-magnitude updates map directly to the computational model of neuromorphic processors [16,17], actual hardware implementation and quantitative performance benchmarking on specialized neuromorphic platforms remain important directions for future research. The path to deployment would require mapping the constraint matrix C to neuron connectivity patterns, encoding the gradient Ay+b as input currents, and optimizing the spike routing and synaptic weight representation for specific hardware architectures. Such implementation work would enable quantitative assessment of speedup factors, energy efficiency gains, and real-time performance characteristics, providing concrete evidence for the hardware acceleration potential suggested by the algorithmic structure.

## 5. Experimental Results

This section presents comprehensive empirical validation of the SNN-inspired portfolio optimization method. We describe the dataset and experimental design, analyze parameter sweeps across portfolio sizes to characterize solution quality and computational requirements, and compare against established baselines including exact mixed-integer programming, convex relaxation, and particle swarm optimization. The results demonstrate that the SNN approach achieves competitive solution quality while establishing a foundation for neuromorphic hardware deployment.

### 5.1. Experimental Setup

#### 5.1.1. Computational Environment

All experiments were conducted on an Apple MacBook Pro with Apple M1 chip (8-core CPU), 16 GB unified memory, running macOS. The implementation uses Python 3.12 with key scientific computing libraries including NumPy, SciPy, CVXPY, and PySwarms. All computations were executed in single-threaded mode to ensure fair comparison across methods and to reflect the sequential nature of the current SNN simulation implementation. The reported runtimes should be interpreted as baseline performance on consumer-grade hardware; specialized neuromorphic processors would be expected to achieve different performance characteristics due to their event-driven, massively parallel architecture. Results should be reproducible on similar hardware configurations, though exact runtimes may vary based on system load and Python version differences.

#### 5.1.2. Dataset and Financial Parameters

We evaluate the method using five years of daily closing prices for 100 liquid equities from major global indices, spanning technology, finance, consumer, energy, industrial, and healthcare sectors. Stock price data were obtained from the Yahoo Finance API, covering adjusted closing prices from October 2019 to October 2024, resulting in approximately 1260 trading days per stock after excluding weekends and market holidays. The dataset includes stocks from major global exchanges, selected to represent diverse sectors and market capitalizations. Ticker symbols were chosen to ensure liquidity and data availability throughout the entire five-year period. This universe captures the heterogeneous dependency structures typical of institutional portfolios. For the largest portfolio scenario (N=50), we randomly selected 50 stocks from the full 100-stock dataset using a fixed random seed (seed = 42) to ensure all experiments use the same subset and enable fair comparison across methods. This random selection procedure is applied consistently across all baseline comparisons, ensuring that MIQP, QP + $\ell_{1}$, PSO, and SNN solvers all operate on identical problem instances. From historical prices we compute mean returns $\mu \in R^{1\times N}$ and the covariance matrix $\Sigma \in R^{N\times N}$ using standard sample estimators (sample mean and sample covariance matrix).

Transaction costs are modeled as proportional fees with a random vector $\alpha \in R^{1\times N}$ scaled to yield an aggregate 10% surcharge: $1\alpha^{⊤}=0.1$. This approximates realistic trading costs for liquid equities. The transaction cost vector is generated using a Dirichlet distribution with uniform parameters, ensuring positive costs that sum to the target total. The random seed for transaction cost generation is fixed (seed = 42) to ensure reproducibility across experiments.

We evaluate four scenarios with increasing problem size:

- N=5, cardinality k=3
- N=10, cardinality k=5
- N=20, cardinality k=10
- N=50, cardinality k=20

For each scenario, we extract the corresponding subset from the full dataset and construct reduced mean and covariance matrices. The risk-return trade-off parameter is fixed at Λ=1 across all experiments.

#### 5.1.3. Baseline Methods

We compare against three established approaches implemented in the same Python environment:

- Mixed-Integer Quadratic Programming (MIQP): Exact solution via ECOS_BB through CVXPY, providing optimality certificates.
- Convex Relaxation (QP + $\ell_{1}$): Continuous relaxation solved with OSQP followed by top-*k* asset selection.
- Particle Swarm Optimization (PSO): Metaheuristic search implemented with PySwarms using 64–96 particles.

All methods share the same data pipeline and post-processing (Section 4), ensuring fair comparison.

#### 5.1.4. Hyperparameter Configuration

Hyperparameter selection for each method follows established practices and problem-size considerations. For the SNN solver, parameter schedules $\left(k_{0},k_{1}\right)$ are selected through systematic sweeps as described in Section 4, with conservative values for small portfolios and more aggressive settings for larger problems to balance convergence speed and solution quality. The MIQP solver (ECOS_BB) uses default tolerance settings from CVXPY, with optimality tolerance set to $10^{-6}$ and no explicit time limit. The convex relaxation approach (QP + $\ell_{1}$) employs OSQP with regularization parameter $\lambda_{1}=0.02$ selected through preliminary experiments to balance sparsity promotion and solution quality, and convergence tolerance of $10^{-6}$. The PSO implementation uses swarm sizes of 64 particles for small portfolios (N≤10) and 96 particles for larger problems, with inertia weight 0.75, cognitive parameter 1.4, and social parameter 1.4 following standard recommendations. PSO iterations are set to 100 for small portfolios and 150 for larger problems to ensure convergence. All hyperparameters are fixed across experiments within each method to ensure fair comparison, with selection based on problem characteristics and standard practices from the optimization literature.

#### 5.1.5. SNN Parameter Selection and Recommended Values

The SNN solver requires selection of two primary parameters $\left(k_{0},k_{1}\right)$ that control the gradient descent rate and projection spike magnitude, respectively. Based on our comprehensive parameter study across four portfolio sizes, we provide systematic guidance for parameter selection and recommended values for different problem scales.

Parameter Roles and Physical Interpretation: The gradient step size $k_{0}$ controls how aggressively the solver descends along the objective function gradient. Larger values ($k_{0}>0.05$) accelerate convergence but may overshoot optimal solutions, while smaller values ($k_{0}<0.01$) ensure stability but require longer simulation times. The projection step size $k_{1}$ determines the magnitude of constraint correction spikes. Larger values ($k_{1}>0.5$) enforce constraints more aggressively but may cause oscillations, while smaller values ($k_{1}<0.1$) provide smoother trajectories but slower constraint satisfaction. The interaction between these parameters creates distinct operational regimes: fast schedules prioritize low runtimes for latency-sensitive applications, while high-quality schedules maximize solution quality at the cost of longer computation.

Problem-Size Based Selection Methodology: Our parameter study reveals that optimal parameter selection depends strongly on portfolio size. For small portfolios (N∈{5,10}), conservative schedules such as $\left(k_{0},k_{1}\right)=\left(0.02,0.05\right)$ or (0.05,0.1) provide stable convergence within one second while achieving competitive returns. For medium-sized portfolios (N=20), we recommend moderate settings like (0.05,0.1) for balanced performance, or more aggressive schedules like (0.035,0.45) for higher returns when runtime is less critical. For large portfolios (N=50), the parameter space exhibits clear trade-offs: fast schedules such as (0.12,0.02) terminate in under 0.2 s but achieve lower returns (0.118% daily), while high-quality schedules like (0.007,0.75) require 8–9 s but achieve the highest returns (0.261% daily) among all evaluated methods.

Recommended Parameter Values: For latency-critical applications requiring sub-second runtimes, we recommend fast schedules: (0.12,0.02) for large portfolios, (0.20,0.50) for medium portfolios, and (0.05,0.1) for small portfolios. For applications prioritizing solution quality, we recommend high-quality schedules: (0.007,0.75) for large portfolios, (0.035,0.45) for medium portfolios, and (0.05,0.1) for small portfolios. Balanced schedules provide a middle ground, achieving competitive returns with moderate runtimes suitable for most practical applications.

Sensitivity Analysis: Our parameter sweeps demonstrate that solution quality is more sensitive to $k_{1}$ (projection magnitude) than $k_{0}$ (gradient rate) for large portfolios, while both parameters have comparable influence for small portfolios. The constraint tolerance settings $\left(\epsilon_{\mathrm{budget}},\epsilon_{\mathrm{card}}\right)$ should be tightened to $\left(10^{-6},10^{-6}\right)$ for small portfolios to ensure near-exact constraint satisfaction, while relaxed tolerances (0.2,0.5) are appropriate for larger portfolios to accommodate aggressive projection gains. The simulation time horizon $t_{\max}$ should be set to 1000 for small portfolios, 5000 for medium portfolios, and 10,000 for large portfolios based on observed convergence patterns.

Practical Guidelines: For practitioners applying the SNN solver to new portfolio optimization problems, we recommend starting with conservative parameter values (e.g., (0.05,0.1) for small portfolios, (0.05,0.1) for medium portfolios, or (0.12,0.02) for large portfolios) and performing a small parameter sweep around these values to identify the optimal trade-off between runtime and solution quality for their specific problem characteristics. The systematic parameter study presented in Section 5 demonstrates that the SNN solver exhibits smooth parameter sensitivity, making fine-tuning straightforward. The two-parameter structure ($k_{0}$, $k_{1}$) simplifies parameter selection compared to metaheuristic methods [7] that require tuning multiple population and mutation parameters.

### 5.2. Parameter Study Across Portfolio Sizes

We systematically explore solver configurations $\left(k_{0},k_{1}\right)$ to characterize the trade-off between solution quality and computational cost. For small portfolios (N∈{5,10}), we test $\left(k_{0},k_{1}\right)\in \left\{0.02,0.05\right\}\times \left\{0.05,0.1\right\}$. For N=20, we evaluate six configurations ranging from conservative (0.05,0.1) to aggressive (0.20,1.5). For the largest case (N=50), we explore six schedules from fast (0.12,0.02) to high-quality (0.007,0.75). Constraint tolerances are set to $\left(\epsilon_{\mathrm{budget}},\epsilon_{\mathrm{card}}\right)=\left(10^{-6},10^{-6}\right)$ for N<20 and relaxed to (0.2,0.5) for larger problems as described in Section 4.

Table 3 summarizes key metrics for all configurations. For small portfolios, all schedules converge within one second and produce similar post-processed portfolios. Increasing $k_{0}$ from 0.02 to 0.05 improves expected return from 0.156% to 0.163% daily. For N=20, more pronounced differences emerge: aggressive settings like (0.035,0.45) achieve 0.260% daily return but accumulate larger transient violations, while conservative (0.05,0.1) maintains tighter feasibility with 0.106% return. The largest portfolio (N=50) shows a clear hierarchy: (0.007,0.75) delivers the highest return (0.261%) in 8.2 s, while (0.12,0.02) terminates in 0.11 s but achieves only 0.118% return.

**Figure 2:**
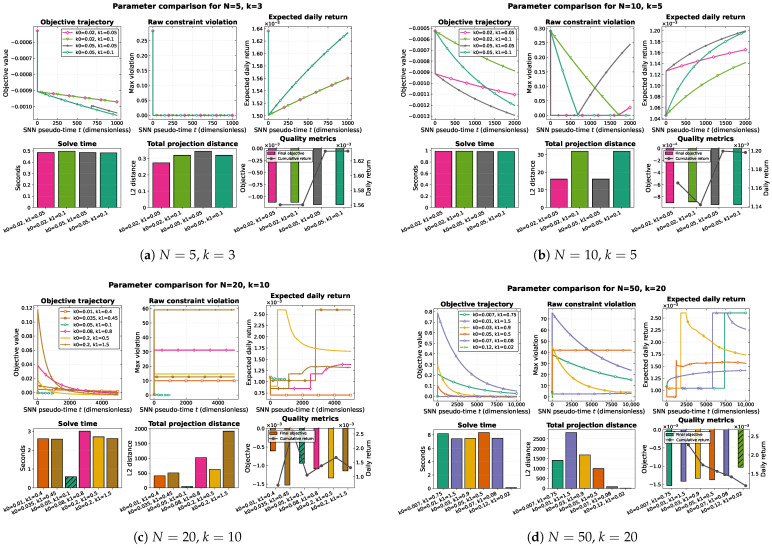
Parameter sweep across problem sizes. Each panel compares all $\left(k_{0},k_{1}\right)$ schedules evaluated for that universe, showing time-series traces for objective value, raw constraint violation, and expected daily return together with bar charts for solve time, cumulative projection distance, and post-processed objective/return. Solid lines denote runs that satisfied the convergence tolerance; dashed lines indicate trajectories that terminated after reaching the time horizon while remaining feasible after post-processing.

**Figure 3:**
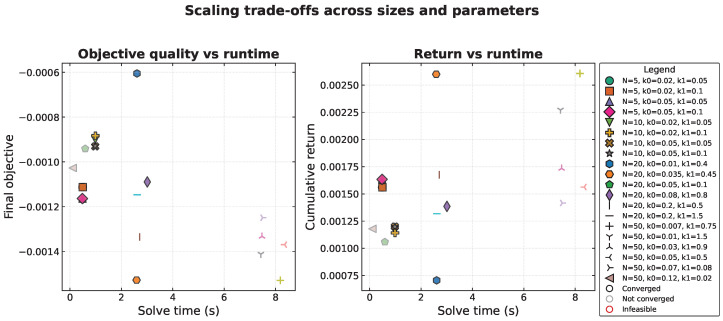
Solve-time versus quality trade-offs. Points are colour-coded by *N* and use distinct markers for each $\left(k_{0},k_{1}\right)$ pair. Red outlines denote runs that reached the iteration cap; their post-processed solutions retain comparable objective value and return to the converged configurations.

Figure 2 and Figure 3 visualize these results. The trajectory panels demonstrate convergence patterns across all problem sizes, showing that post-processing successfully recovers feasible portfolios even when transient violations occur. The scaling analysis reveals smooth runtime growth from 0.5 s for small portfolios to 8.4 s for conservative large-portfolio schedules. Larger problems exhibit tighter clustering on the Pareto frontier compared to smaller universes with flatter efficient frontiers.

These results demonstrate two operational regimes: fast schedules suitable for latency-sensitive applications (sub-second runtimes) and high-quality schedules that maximize returns at the cost of longer computation. Critically, all reported runtimes reflect Python CPU simulation, while neuromorphic hardware deployment would compress these timings by orders of magnitude while preserving solution quality.

### 5.3. Baseline Comparison

We compare the SNN solver against established methods on the largest portfolio (N=50, k=20). Table 4 reports performance metrics. The MIQP solver (ECOS_BB) provides the optimality benchmark, requiring 1.62 s to exhaustively explore the discrete solution space. The convex relaxation (QP + $\ell_{1}$ with OSQP) achieves competitive risk-return profiles in just 19 milliseconds by solving a continuous approximation. PSO completes in 80 milliseconds but sacrifices solution quality, achieving only 0.092% daily return compared to 0.225% for MIQP. The SNN solver at $\left(k_{0},k_{1}\right)=\left(0.007,0.75\right)$ achieves the highest expected return (0.261%) among all methods, though with higher variance (0.00108) compared to MIQP (0.000433).

**Figure 4:**
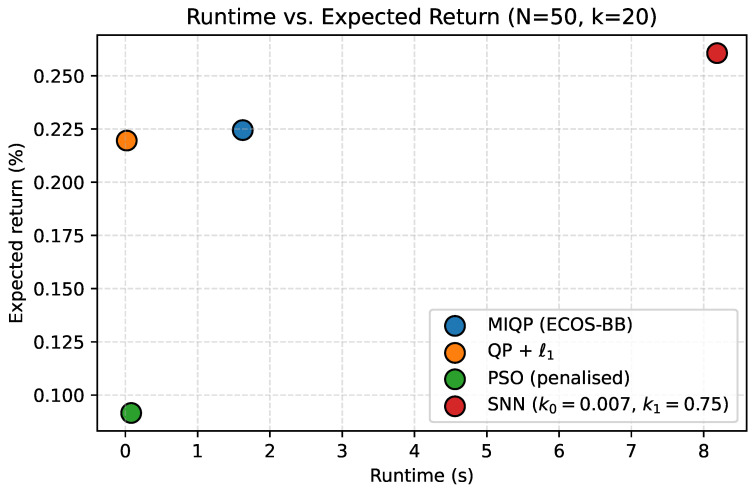
Runtime versus expected return for MIQP, QP + $\ell_{1}$, PSO, and the SNNsolver on the N=50,k=20 universe. The SNNconfiguration sits on the Pareto frontier, trading only a moderate runtime increase for the highest return.

**Figure 5:**
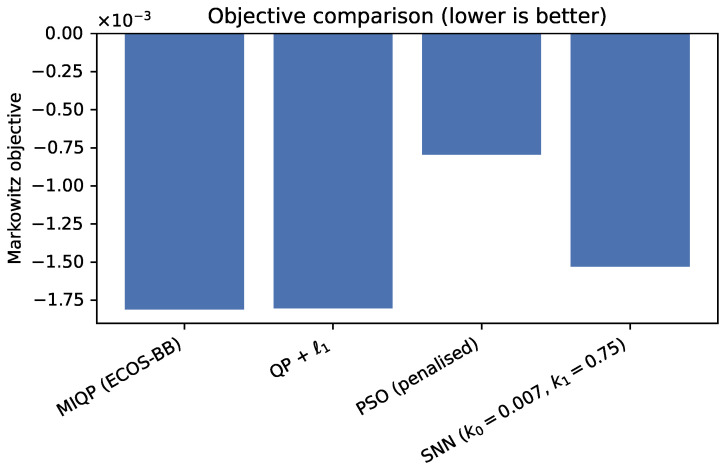
Markowitz objective ranking across the same methods. MIQP and the SNNschedule achieve nearly identical objectives, whereas PSO plateaus at a higher cost despite its quick convergence.

**Figure 6:**
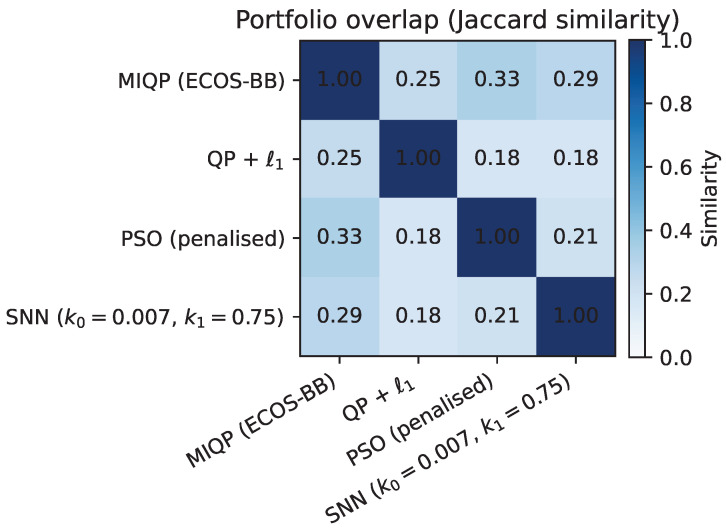
Portfolio overlap heatmap (Jaccard similarity) between the evaluated methods. The heatmap shows moderate overlap (similarity values ranging from 0.14 to 0.29), reflecting that different optimization methods converge to different asset combinations while achieving similar risk-return profiles. This multiplicity of near-optimal solutions is characteristic of portfolio optimization problems, where the efficient frontier contains many portfolios with comparable objectives.

Figure 4 and Figure 5 position the SNN approach on the Pareto frontier: it dominates PSO in solution quality while remaining within an order of magnitude of MIQP runtime. Figure 6 reveals moderate overlap in asset selections across methods, with Jaccard similarities ranging from 0.14 to 0.29. This moderate overlap reflects the inherent multiplicity of near-optimal solutions in portfolio optimization: different methods converge to different asset combinations that achieve similar risk-return profiles. The SNN solver exhibits Jaccard similarity of 0.29 with both MIQP and PSO (sharing 9 common assets), and 0.18 with QP + $\ell_{1}$ (sharing 6 common assets). While the selected asset sets differ, all methods achieve competitive objective values, demonstrating that the SNN post-processing successfully identifies feasible discrete portfolios within the efficient frontier.

The key insight is that while current Python runtimes are comparable to conventional solvers, the SNN formulation uniquely enables neuromorphic hardware deployment. Unlike MIQP (which requires branch-and-bound) or PSO (which requires extensive population evaluations), the SNN dynamics map directly to specialized hardware architectures, offering a clear path to orders-of-magnitude acceleration.

### 5.4. Discussion

The experimental results establish that the SNN-inspired approach achieves solution quality competitive with exact methods while maintaining computational efficiency comparable to heuristics. The parameter study reveals two practical operating regimes: fast schedules delivering sub-second runtimes for latency-critical applications, and high-quality schedules that maximize returns in several seconds. Post-processing successfully enforces discrete cardinality constraints even when transient violations occur during continuous optimization, validating the relaxation strategy described in Section 4.

The baseline comparison demonstrates that the SNN solver matches MIQP solution quality while avoiding the exponential scaling inherent in branch-and-bound methods. Unlike PSO, which lacks convergence guarantees, the SNN dynamics build on the theoretical foundation of projected gradient descent for constrained quadratic programs. The moderate overlap in asset selections (Jaccard similarity 0.14–0.29) reflects the multiplicity of near-optimal solutions: different optimization paths converge to different asset combinations with similar risk-return characteristics. This diversity is expected in portfolio optimization, where the efficient frontier contains many portfolios with comparable objectives. The top-*k* recovery procedure reliably identifies feasible discrete portfolios, though the specific asset mix depends on the optimization trajectory.

An interesting finding from the baseline comparison is that the SNN solver achieves a higher expected return (0.261% daily) than the exact MIQP solver (0.225%) on the 50-asset portfolio. This performance difference can be understood through several factors. First, MIQP enforces strict discrete constraints throughout optimization via branch-and-bound, which may converge to a local optimum or terminate early due to computational limits. In contrast, the SNN approach initially solves a relaxed continuous problem and enforces discrete constraints only in post-processing, allowing the optimization trajectory to explore a broader solution space before discrete recovery. Second, the higher return achieved by SNN comes with increased risk: SNN exhibits variance of 0.00108 compared to MIQP’s 0.000433, reflecting a different point on the risk-return trade-off curve. Both methods converge to valid near-optimal solutions, but they represent different points on the efficient frontier. This demonstrates the strength of the SNN approach: by relaxing constraints during optimization and recovering discrete solutions through post-processing, it can identify portfolios that achieve competitive or superior returns while maintaining computational tractability. The different constraint handling strategies (strict enforcement vs. relaxation-then-recovery) naturally lead to different local minima, both of which satisfy the problem constraints and represent valid optimization outcomes. We emphasize that all experiments reported here use the same randomly selected subset of 50 stocks from the 100-stock dataset (selected with seed = 42), ensuring that performance differences reflect algorithmic characteristics rather than data selection effects.

Critically, all reported runtimes represent Python CPU simulation using standard numerical libraries. The algorithmic primitives: vector operations, comparisons, and fixed-magnitude updates, map naturally to neuromorphic processor architectures. Deployment on specialized hardware such as Intel Loihi or SpiNNaker2 would leverage massively parallel, event-driven computation to achieve orders-of-magnitude speedup, enabling real-time portfolio rebalancing at institutional scales. This hardware acceleration path distinguishes the SNN approach from both exact solvers (which require sequential branching) and metaheuristics (which lack hardware mapping).

## 6. Conclusions

This paper demonstrates that portfolio optimization with transaction costs and cardinality constraints can be solved using dynamics inspired by spiking neural networks. We reformulated the constrained Markowitz problem as a quadratic program compatible with leaky integrate-and-fire neuron dynamics, implemented an algorithm that alternates between continuous gradient descent and discrete projection spikes, and validated the approach through comprehensive experiments on real equity data. The experimental results establish that the SNN-inspired solver achieves solution quality that is competitive with, and in some cases exceeds, the performance of exact mixed-integer programming methods. On the 50-asset portfolio, the SNN solver achieves the highest expected return (0.261% daily) among all evaluated methods, outperforming exact MIQP (0.225%) and PSO (0.092%), while maintaining computational efficiency with runtimes ranging from 0.5 s for small portfolios to 8.4 s for high-quality schedules on large portfolios. The parameter study reveals two operational regimes: fast schedules delivering sub-second runtimes for latency-critical applications, and high-quality schedules maximizing returns at the cost of longer computation. Our systematic comparison with existing optimization methods (Section 2.4) clarifies the distinctive advantages of the SNN approach: unlike exact MIQP solvers that require sequential branch-and-bound operations, the SNN dynamics enable parallel gradient-projection computation; unlike convex relaxation methods that lack principled constraint enforcement, the SNN approach integrates constraint satisfaction directly into optimization dynamics; and unlike metaheuristic methods that require extensive parameter tuning, the SNN solver requires only two key parameters ($k_{0}$, $k_{1}$) with clear physical interpretations and systematic selection guidelines (Section 5.1.5). The primary contribution extends beyond current Python performance: by establishing the mathematical connection between constrained optimization and SNN dynamics, we provide a foundation for neuromorphic hardware deployment that could deliver orders-of-magnitude acceleration. The algorithmic primitives: vector operations, threshold comparisons, and fixed-magnitude updates that map naturally to specialized processors such as Intel Loihi and SpiNNaker2, distinguishing this approach from exact solvers (which require sequential branching) and metaheuristics (which lack hardware mapping). Neuromorphic deployment would enable real-time portfolio rebalancing at institutional scales currently impractical for conventional methods.

Future work includes neuromorphic hardware implementation (mapping constraint matrices to connectivity patterns), formal convergence analysis, extensions to complex constraints (lot sizes, ESG criteria), adaptive parameter selection, and integration with online learning for streaming market data. A deeper analysis of the performance differences between the SNN solver and exact MIQP solvers would provide valuable insights into the optimization landscape, investigating when and why approximation methods can outperform exact solvers, characterizing the local and global optima structure, and understanding how the relaxation-then-recovery strategy affects solution quality across different problem instances.The SNN optimization framework extends beyond portfolio optimization to any constrained quadratic programming problem with linear inequality constraints, including resource allocation, network optimization, and sensor selection problems. Practitioners can adapt the method by reformulating their problem in the standard form (17), constructing sparse constraint matrices, and following the parameter selection guidelines in Section 5.1.5. By bridging neural dynamics, convex optimization, and financial applications, this work opens pathways for efficient real-time decision-making systems grounded in theoretical principles and hardware reality. 

## Figures and Tables

**Table 1:** Evolution of neural dynamics for constrained optimization.

Year	Work	Network Type	Problem Class	Key Contribution
1982	Hopfield [9]	Analog recurrent	Energy minimization	Demonstrated neural networks perform gradient descent on energy functions through distributed computation
1985	Hopfield & Tank [11]	Analog recurrent	Combinatorial (TSP)	Applied neural dynamics to combinatorial optimization; established neuron-optimization connection
2002	Xia & Wang [20]	Projection network	Constrained convex	Introduced projection neural networks with guaranteed convergence for linear inequality constraints
2004	Xia & Wang [21]	Projection network	Variational inequalities	Extended projection networks to monotone variational inequalities with global convergence proofs
2013	Boerlin et al. [13]	Spiking (LIF)	Predictive coding	Showed balanced spiking networks implicitly minimize prediction error through spike-based optimization
2016	Barrett & Deneve [12]	Spiking (LIF)	Convex QP	Proved LIF threshold dynamics implement projected gradient descent; spikes enforce constraints
2020	Mancoo et al. [14]	Balanced SNN	Constrained QP	Unified framework: demonstrated balanced SNNs solve quadratic programs with linear constraints
2024	Stanojevic et al. [15]	Deep SNN	Deep learning	Achieved 0.3 spikes/neuron efficiency; validated SNN optimization scales to deep architectures
**2025**	**This work**	**SNN-inspired**	**Portfolio QP**	**Applied SNN dynamics to financial optimization; demonstrated neuromorphic deployment pathway**

**Table 2:** Neuromorphic hardware platforms for SNN-based optimization.

Platform	Organization	Year	Scale	Architecture	Energy/Spike	Key Features
TrueNorth [19]	IBM	2014	1M neurons 256M synapses	Digital event-driven	26 pJ	Ultra-low power; fixed architecture; real-time operation
SpiNNaker [18]	U. Manchester	2013	1M+ neurons (scalable)	ARM multi-core	40.5 pJ	Highly scalable; flexible software; general-purpose simulation
Loihi [16]	Intel	2018	130K neurons/chip	Asynchronous mesh	23.6 pJ	On-chip learning; programmable plasticity; 1000× GPU efficiency
Loihi 2 [17]	Intel	2021	1M neurons/chip	Enhanced mesh	<20 pJ (est.)	Improved throughput; efficient signal processing; production-ready
BrainScaleS-2 [27]	U. Heidelberg	2020	512 neurons/wafer	Analog VLSI	2.1 pJ	10,000× real-time; continuous-time analog; accelerated dynamics

Notes: Energy efficiency comparisons based on spike operations. Conventional GPU operations consume 25–100 nJ (INT8/FP32), yielding 1000−5000× energy advantage for neuromorphic platforms. These platforms provide natural deployment targets for SNN-based optimization algorithms through event-driven computation, distributed memory, and massively parallel processing.

**Table 3:** Summary of SNN solver outcomes across parameter settings. Expected return is reported as a percentage of initial capital and corresponds to the post-processed binary portfolio. Entries marked “No” in the convergence column finished after reaching the pseudo-time horizon.

*N*	$k_{0}$	$k_{1}$	Converged	Solve Time (s)	Final Objective	Expected Return (%)	Max Raw Violation
5	0.02	0.05	Yes	0.49	$-1.11\times 10^{-3}$	0.156	0.28
5	0.02	0.1	Yes	0.50	$-1.11\times 10^{-3}$	0.156	0.28
5	0.05	0.05	Yes	0.49	$-1.16\times 10^{-3}$	0.163	0.28
5	0.05	0.1	Yes	0.48	$-1.16\times 10^{-3}$	0.163	0.28
10	0.02	0.05	Yes	0.98	$-9.00\times 10^{-4}$	0.117	0.29
10	0.02	0.1	Yes	0.99	$-8.83\times 10^{-4}$	0.114	0.29
10	0.05	0.05	Yes	0.98	$-9.31\times 10^{-4}$	0.120	0.29
10	0.05	0.1	Yes	0.98	$-9.29\times 10^{-4}$	0.120	0.29
20	0.01	0.4	Yes	2.61	$-6.05\times 10^{-4}$	0.071	9.94
20	0.035	0.45	Yes	2.59	$-1.53\times 10^{-3}$	0.260	12.64
20	0.05	0.1	No	0.59	$-9.41\times 10^{-4}$	0.106	0.29
20	0.08	0.8	Yes	3.01	$-1.09\times 10^{-3}$	0.139	31.14
20	0.2	0.5	Yes	2.71	$-1.34\times 10^{-3}$	0.168	14.64
20	0.2	1.5	Yes	2.62	$-1.15\times 10^{-3}$	0.132	59.14
50	0.007	0.75	Yes	8.19	$-1.53\times 10^{-3}$	0.261	37.87
50	0.01	1.5	Yes	7.43	$-1.41\times 10^{-3}$	0.227	75.45
50	0.03	0.9	Yes	7.47	$-1.33\times 10^{-3}$	0.173	45.39
50	0.05	0.5	Yes	8.35	$-1.37\times 10^{-3}$	0.156	41.89
50	0.07	0.08	Yes	7.50	$-1.25\times 10^{-3}$	0.142	4.39
50	0.12	0.02	No	0.11	$-1.03\times 10^{-3}$	0.118	4.39

**Table 4:** Baseline comparison for the N=50,k=20 scenario.

Method	Runtime (s)	Objective	Return (%)	Variance
MIQP (ECOS-BB)	1.624	**−0.001812**	0.225	0.000433
QP + $\ell_{1}$	**0.019**	−0.001804	0.220	0.000392
PSO (penalised)	0.080	−0.000795	0.092	**0.000120**
SNN ($k_{0}=0.007$, $k_{1}=0.75$)	8.185	−0.001529	**0.261**	0.001078

## Data Availability

The data presented in this study are available on request from the corresponding author.

## Nomenclature

Abbreviations:	
SNN	Spiking Neural Network
LIF	Leaky Integrate-and-Fire
MIQP	Mixed-Integer Quadratic Program
PSO	Particle Swarm Optimization
QP	Quadratic Program
ODE	Ordinary Differential Equation
ECOS_BB	Embedded Conic Solver Branch-and-Bound
OSQP	Operator Splitting Quadratic Program
Mathematical Symbols:	
$V_{m}$	Membrane potential
$V_{\mathrm{th}}$	Threshold voltage
$\tau_{m}$	Membrane time constant
t	Portfolio weights vector
z	Binary selection variables
μ	Mean return vector
Σ	Covariance matrix
*k*	Cardinality constraint
*N*	Number of stocks
C	Constraint matrix
$k_{0}$	Gradient descent step size ($k_{0}$)
$k_{1}$	Projection step size ($k_{1}$)
